# Are We in an Ethical Dilemma in Aesthetic Medicine?

**DOI:** 10.1111/jocd.70260

**Published:** 2025-06-04

**Authors:** Kamran Izhar Qureshi, Franco Vercessi, Hina Farooq, Rolf Soenchen, Mughese Amin, Izza Chattha, Fatima Tariq, Shagufta James, Haadia Qayum Khan

**Affiliations:** ^1^ Antiage Lifestyle Clinic Gulberg Lahore Pakistan; ^2^ Via Vincenzo Monti Milano Italy; ^3^ Koster Clinic Dubai UAE; ^4^ Amin Clinic Bahawalpur Pakistan

**Keywords:** aesthetic effects, aesthetic medicine, aesthetic surgery, cosmeceuticals

## Abstract

**Introduction:**

The business, medical community, and patients are under tremendous strain due to advancements in aesthetic medicine and the rising demand for cosmetic therapies. It is impossible to overlook the financial benefits. Social media may have both beneficial and detrimental effects in the marketing of aesthetic services. Several ethical considerations need to be carefully considered, such as beneficence and maleficence as well as the rights of the patient. Is monetary gain the main goal? There must be an answer to this query. Medicine requires a high standard of professionalism and ethics, and the same rule applies for aesthetic medicine and surgery.

**Methods:**

This is a questionnaire‐based study targeting the practicing doctors and the general population. A sample size of 100 per segment was selected. The participants of the study were from various ethnicities from around the globe.

**Results:**

The results of the study clearly implicate that most of the participants agree that Aesthetic Medicine is facing an ethical dilemma. Serious measures need to be taken to regularize the field of aesthetics in the form of training of the doctors, strict measures by the regulators, and harmonization of the marketing through social media.

**Conclusion:**

Aesthetic medicine and surgery have become a commodity rather than a recognized medical specialization due to their high evolution index and commercial incentives. This study argues that operating based on market categories can lead to a loss of focus on patients' true needs. If aesthetic medicine doesn't recognize itself as part of ethical medicine, it may become a part of the beauty business, prioritizing profit over public needs.

## 1 Introduction

Innovations in aesthetic medicine and the growing demand for aesthetic procedures have put immense pressure on the industry, medical community, and clients. The commercial gains cannot be ignored. Social media has both positive and negative influences. Clinics and doctors have gotten carried away with the use of these mediums and are behaving more as entertainers than professionals. Careful thought must be given to several ethical issues, including beneficence and maleficence as well as the patient's rights. Is money the primary objective or not? This question needs to be answered.

The ever going debate about commercialism in medicine is something we all may have heard about [1]. Medicine is at a level where ethics and professionalism must be above par. An offshoot of conventional medicine is the field of aesthetics, where the boundaries are yet to be defined. Undoubtedly, aesthetic medicine is one of the fastest growing industries with a very high evolution index. With the increasing demand, the need for ethical practice has become more important [2]. This paper looks at the field of Aesthetic Medicine considering the nonethical practices being carried out.

## 2 Materials and Methods

Though there are many papers that talk about the ethical aspect of aesthetic medicine, this is the first study that has reached out to the doctors and general population to take their view on this sensitive topic. Two sets of population were selected for the study: Doctors and general population. A questionnaire for each population subset was designed after thorough deliberation between the study investigators. The questionnaire was circulated in the medical community practicing aesthetic medicine and the general population through social media, WhatsApp groups, and other channels. The sample size was set at 100 for each group.

## 3 Validation of the Questionnaire

Face validation and pilot testing were used to validate the questionnaire. For the face validation, the questionnaire was sent to a group of experts comprising doctors working in the field of aesthetics and patients/clients visiting aesthetic clinics. The only concern raised was about the length of the questionnaire. The questions were found pertinent and focused. A pilot was done on 10 responses for each questionnaire, and the results were made part of the actual survey.

## 4 Analysis

### 4.1 Doctors Perspective

The survey responses from a sample of 110 doctors from across the globe belonging to different ethnicities for their perspective on the ethical dilemma of aesthetic medicine reveal a fascinating conflict in the field. According to the responses, 76.4% of doctors believe we are in an ethical dilemma. The same question was asked at the end of the survey, asking the doctors if they felt that they were still in an ethical dilemma after filling out the survey, and the number rose to 82.7%. This increase from 76.4% to 82.7% implies that many doctors are aware of an underlying conflict.

89.1% of respondents also responded that physiotherapists, pharmacists, or non‐medical doctors should not be allowed to perform injectables. Additionally, when the doctors were asked if dentists should be allowed to treat skin problems and body areas other than the face 91.8% said ‘No’. This statistical evidence urges medical organizations to reconsider the qualifications and the scope of work they are allowed to do. Additionally, if we look at ensuring patient well‐being over financial gain 74.5% of doctors agreed that it was vital as patient care should be a medical doctor's top priority. 33.6% of doctors also agree that financial incentives in aesthetic medicine led to overtreatment of patients. These findings point to a serious lack of ethical transparency and patient education, which can further damage patient‐physician confidence.

Moreover, only 14.5% of respondents are extremely confident that patients are provided with sufficient information to make informed decisions and only 20% feel that they are ‘often’ provided with accurate and unbiased information about procedures at aesthetic clinics. However, results show that 69.1% believe that the responsibility to ensure the risks and benefits are fully understood before treatment lies with both the doctor and the patient. Lastly, 76.4% agree that there is a race among clinics to be the first one to offer a procedure (Me‐first Syndrome). With the “Me‐First Syndrome”, clinics scramble to offer innovative procedures before others. Although innovation is essential, this kind of competition may jeopardize patient safety and result in hastily administered, inadequately studied medicines. This leads us to the conclusion that aesthetic practitioners are both creating and curing insecurities and 63% of doctors agree.

If we add marketing and its regulations to the crux, 65.5% ‘strongly agree’ and ‘agree’ that regulations should be enforced to control how aesthetic procedures are marketed on social media. Over 50% also ‘agree’ that before and after images used in marketing can cause unrealistic expectations.

A significant 91.8% of physicians were concerned that a three to five‐day certification program is not enough to perform an aesthetic procedure and believe ongoing mentorship or supervised practice should be required after completing the initial course. Additionally, most responders support continued mentorship or supervised practice to guarantee proficiency and patient safety. This emphasis on prolonged, systematic training highlights the necessity of a strong foundation in evidence‐based practices rather than a rapid, cursory introduction to the area. Over 30% of doctors disagreed when they were asked if evidence‐based medicine was being followed, and this proves to be a cause of concern as medicinal credibility and following evidence‐based practices is essential.

The 85.5% who think that specialized training in recognizing and treating Body Dysmorphic Disorder (BDD) is crucial also suggest a strong call for integrating mental health awareness into aesthetic practices as a safeguard against treatments that could harm vulnerable patients. However, according to 63% of respondents, aestheticians might be making people more anxious rather than less, indicating that the lines separating medical care from cosmetic procedures are becoming hazier. In this tendency, consumerism may take precedence over patient identity and well‐being, potentially transforming aesthetic medicine into a service‐oriented profession.

According to 87.5% of physicians, the pressure to promote services on social media only serves to intensify this trend, possibly pushing medical practitioners in the direction of a beauty salon‐style model. This model puts appearance and financial gain ahead of patient care and evidence‐based treatment. To distinguish aesthetic medicine from non‐medical beauty treatments, there is a movement for it to be acknowledged as a specialized medical discipline; 44.5% of respondents support this approach. Given the possibility of being viewed as service providers rather than medical professionals, doctors appear to be concerned about losing their professional integrity.

Please refer to Appendix S1 for the complete questionnaire and results.

### 4.2 General Population

A possible ethical dilemma is highlighted by the survey's results, which were obtained from a sample of 114 members of the general population. The survey also revealed significant ethical concerns and opposing viewpoints within the area of aesthetic medicine. There appears to be broad skepticism as a sizable percentage of respondents (59.3%) think that there is an ethical dilemma in aesthetic medicine, while 30.1% are not sure. The same question was also asked at the end of the survey, and after filling the survey, the percentage grew to 73.5% of individuals believing we were in an ethical dilemma, indicating an increase in awareness and reflection. More than 60% of respondents said that injectable procedures should never or very seldom be administered by physiotherapists, pharmacists, dentists, and non‐medical doctors, which is partially due to the increase of non‐medical practitioners doing these operations. According to this, concerns about patient safety are raised by the increasing number of non‐medical professionals performing these therapies.

There is a clear preference for specialized care, as evidenced by the majority's (92.9%) opposition to dentists treating areas outside the face, implying the importance of specialized care. Additionally, 84.8% of respondents think that clinics have a competitive “Me‐First Syndrome” to be the first to offer new procedures, thereby putting market competition ahead of patient safety. 25.7% of the population is also ‘likely’ to try a new aesthetic service being offered by a clinic, emphasizing the use of novelty in decision making. Additionally, from the perspective of clients over 45% are neutral about whether doctors follow evidence or not implying an absence of medicinal evidence. Additionally, the same percentage is unsure if they are even provided with sufficient informed decisions and a striking 12.4% believe that they are not informed. However, 61.9% also believe that this responsibility to ensure risks and benefits completely before the treatment lies with both the doctor and the patient highlighting the lack of awareness from the patient's end. 60.2% of patients also always ask the practitioner about the products being used and the procedure showing patient awareness. While choosing a clinic about 77% of the population looks at the experience of the doctor while 69% look at the qualification implying the importance of the doctor and his experience. 78.8% are also aware that not all practitioners in aesthetic medicine are medical doctors reflecting a gap in the understanding of the public about the credentials of their medical practitioner. Lastly, 68.1% also feel that aesthetic practitioners are both helping cure insecurities and are creating them showing that there is both good and harm being done in the process of aesthetic medicine.

Many respondents (77%) believe that patient well‐being should come before financial incentives, yet some (47.8%) also believe that financial incentives lead to overtreatment. Since about 36.3% of respondents think “before‐and‐after” photos can create irrational expectations, this is consistent with worries about marketing strategies. This worry also extends to the perceived need for regulation; 55.8% of respondents strongly favor stricter guidelines for the promotion of aesthetic operations on social media. Additionally, 40.7% believe modern beauty standards are defined by society, while 31% believe that it is defined by the fashion and beauty industries which play an active role on social media and are thus indirectly influencing the public. 42.5% ‘agree’ and 17.7% ‘strongly agree’ that social media pressures you to alter your appearance or use filters.

Furthermore, over 50% of the general population is not familiar with body Dysmorphic disorder (BDD) and is 60.2% neutral about whether aesthetic procedures should be offered to patients with BDD, which is a cause of concern. Additionally, 61.9% of respondents believe that both the patient and the doctor bear responsibility, indicating a desire for openness and well‐informed decision‐making. However, there is doubt over social media's representations of results, and only 22.1% of respondents think clinics often offer objective information. The influence of social media and contemporary beauty standards generates extra ethical considerations, even as credentials and expertise are appreciated when choosing a practitioner, as demonstrated by linked questions.

Overall, the data indicates that although aesthetic medicine has chances for individual improvement, to preserve credibility and patient safety, it must negotiate a challenging terrain of ethics, trust, and ethical marketing.

Please refer to Appendix S2 for the complete questionnaire and results.

## 5 Discussion

Beauchamp and Childress in the “Principles of Bio‐Medical Ethics” defined a framework that is accepted as a guiding force in making an ethical decision. The four integral principles are:

- Autonomy: respecting the decision‐making capacities of autonomous patients, enabling individuals to make reasoned informed choices.
- Beneficence: balancing benefits of treatments against risks and costs, the healthcare professional should act in a way that benefits the patient.
- Non‐maleficence: Avoid causing harm, even if minimal, but the harm should not be disproportionate to the benefits of the treatment.
- Justice: fairness, entitlement and equality.

Aesthetic Medicine and clinics carry as much responsibility to adhere to these principles as any other form of Medicine.

Clients have the right to choose whether to have a treatment or not, and their informed decision must be respected [3]. However, the source of information is crucial, as social media often provides misleading before and after pictures. It is essential to include both the risks associated with the treatment and alternative options. These principles apply even to aesthetic medicine and surgery, even when the patient is not experiencing any illness. Elective aesthetic treatments involve serious ethical concerns, as they may have long‐term negative effects on body function and health, contradicting the physician's principle of non‐maleficence [4]. In such cases, the patient's right to autonomy may conflict with the physician's commercial gains.

### 5.1 Beneficence or Non‐Maleficence

The principle of beneficence and non‐maleficence is an integral part of ethical medical practice. This applies as much to Aesthetic Medicine as to any other discipline of Medicine. Beneficence means a risk‐benefit analysis leading the healthcare professional to make a decision that benefits the patient. On the other hand, non‐maleficence clearly does not allow even a minimal harm to be done to the patient. In Aesthetic Medicine, where there is a constant conflict between commercial gains, high investments in machines, and lack of evidence, these principles become all the more important.

### 5.2 Who Defines the Beauty Standards? Trends Versus Standards

The question is who is defining the standards of beauty: industry, doctors, or clients ? We all possess an innate sense of beauty that entails balancing the purpose, a capacity to recognize harmony, symmetry, and order, as well as objective standards and subjective feelings. A balance exists between an object's overall appearance and its sections, as well as the different sections in connection to one another. The “unaesthetic” results from the lack of this harmony and could be devastating for the client. Definitions of beauty have been attempted since classical times. Numerous definitions of beauty have been honored at various locations and periods throughout the history of Western civilizations.

According to Greek definitions of beauty, a harmonious balance of facial characteristics was what made a face lovely. They considered the ideal face to be two thirds as large and split into three equal vertical portions.

Beauty criteria are arbitrary. They are always evolving, created by Photoshop studios and preconceived ideas, and often with a commercial gain. They are not a reliable indicator of someone's genuine value and are unrealistic. It's best to concentrate on the traits that are “constants,” the elements that make you, you. What makes you unique as a person and what interests you is not what is shown to you or what you are made to believe [5].

### 5.3 Defining the Scope of Practice—Who Is Allowed to Do What

The practice of aesthetic medicine is often misunderstood, with the term “Dr.” being used by various disciplines, including dentists, pharmacists, lab technologists, vets, physiotherapists, and medics. This confusion can lead to unprofessional practices and misrepresentation of qualifications. The title “Aesthetic Physician” is also misused, misleading the public about the qualifications of professionals. The field of aesthetic medicine is attractive due to its easy‐to‐earn money potential, with certification programs offering a quick start to practice compared to traditional medicine. However, many countries lack proper regulation through accreditation procedures, and many aestheticians lack the necessary training to perform aesthetic operations [6]. The requirement for organized training and accreditation should not be waived for aesthetic medicine practice, as it protects the public from harmful and untested therapies. It is not practical for the cosmetic medicine sector to govern itself, as it may suffer from a lack of professional and ethical standards.

### 5.4 As Doctors Are We Curing Insecurities or Sensitivities or Are We Creating Them?

Clients often ask practitioners about their face, which can be a crucial aspect of building trust. However, many practitioners may reveal flaws or asymmetries that they were unaware of before. This can lead to clients seeing only these flaws in the mirror, which they were previously unaware of. Practitioners may also engage in social gatherings to attract clients, creating insecurities. This can be particularly detrimental for those who cannot afford expensive procedures, as they may collect money to achieve what may not be possible. This can lead to psychological damage [7].

### 5.5 First One to Introduce the Therapy—“The Me‐First Syndrome”

News channel have been notorious in making the claim that they are the first ones to break the news. Today we see the same phenomenon creeping into the world of Aesthetics. Every clinic wants to be the first one to claim that the therapy is introduced first by them. This has created a rat race that may not have any ending. This is also leading to promoting therapies that may not have any scientific basis or evidence as discussed below. The authors have coined this phenomenon as “The Me‐First Syndrome”—a rat race to be the first one to introduce a therapy, not even looking at the evidence. This is leading to false claims and unrealistic expectations being created.

### 5.6 Are We Following Evidence‐Based Medicine?

Applying the pertinent scientific information to the patient's condition based on the patient's values and the clinician's clinical judgment to customize the patient's therapy is made possible by evidence‐based medicine. Using the best available evidence, evidence‐based medicine seeks to improve medical outcomes [8].

Dermatologists and plastic surgeons, followed by a variety of medical specialties, introduced several skin rejuvenation treatment programs (topical creams, skin care products) and skin rejuvenation procedures (such as filler and cosmetic botulinum toxin injections, lasers, light devices, radiofrequency devices, and surgical procedures). Nonmedical professionals, such as beauticians and spa owners, have joined the trend of offering these services. There is little scientific proof to back up the claims made by many of these aesthetic procedures that they can revitalize the skin.

Many examples of such procedures are there. Exosomes, for example, do not have enough data to make a concrete claim as to their benefit, and yet doctors are making claims.

### 5.7 Are Doctors Allowed to Advertise?

The American Medical Association clearly states that “There are no restrictions on advertising by physicians except those that can be specifically justified to protect the public from deceptive practices. A physician may publicize him or herself as a physician through any commercial publicity or other form of public communication (including any newspaper, magazine, telephone directory, radio, television, direct mail, or other advertising) provided that the communication shall not be misleading because of the omission of necessary material information, shall not contain any false or misleading statement, or shall not otherwise operate to deceive.” On the other hand the Medical Council of India prohibits marketing [9].

Different countries have different regulations as far as advertisement is concerned but one thing is universally accepted that advertisement should not be misleading or deceptive. The information provided should be factual and exploitation of the patients' sensitivities and insecurities is not acceptable. Claims of risk‐free therapies and false pretentions of the results cannot be done. False claims and guaranteed results are not allowed to be advertised. Promotional tactics to make a wrong decision should be discouraged. Marketing may be allowed but ‘Responsible Marketing’ is the key.

### 5.8 Recovering the Cost of High‐Priced Machineries?

Putting a good aesthetic clinic requires a lot of investment. Machines are expensive and the cheaper alternatives at times carry risk. As practitioners, it is important to understand that all procedures are not for everyone. Just to recover the cost of the machines, a lot of clinics are doing procedures that may not be needed by the individual.

### 5.9 Body Dysmorphia a Reality That We Conveniently Forget

Recently, social media use has skyrocketed, especially with millennials. The use of filtered images and photo editing has led to a new trend of social media‐induced dissatisfaction with appearance, termed “snapchat dysmorphia” and “selfie dysmorphia” [10]. It is important for practitioners to recognize and understand this trend in addition to knowing how to manage these patients. As clinicians, we have bioethical and professional obligations to educate ourselves on new and relevant trends, ensure adequate patient safety, and advocate for continued consumer education [10].

### 5.10 Social Media and Its Negative Influence

Ateq et al. in a study concluded that “A growing body of evidence suggests that social media may impact mental health in different ways”. This study reveals that heavy use of these platforms is associated with negative appraisals about one's physical appearance, and it fosters one's tendency toward cosmetic surgery, especially among females [11].

Social media platforms like TikTok and Instagram have revolutionized aesthetic medicine, but they also contribute to misinformation and unrealistic beauty standards. Influencer marketing often prioritizes beauty trends over evidence‐based medicine, leading to false endorsements of potentially harmful procedures. This can cause patients to have unrealistic expectations, dissatisfaction, or health consequences. The lack of medicinal credibility further complicates this issue, making it difficult for the general population to differentiate between authentic advice and marketing gimmicks [12].

### 5.11 Recommendations for a Global Regularization

Based on this study, the authors recommend a multi‐pronged approach to regularize the field of aesthetic medicine globally.

1. Standardizing education and training
  ○ Develop comprehensive curricula
  ○ Establish clear qualification criteria
  ○ Promote continuous professional development
2. Implementing a standardized accreditation system
  ○ Develop a global accreditation body
  ○ Establish rigorous standards
  ○ Promote public transparency
3. Strengthening regulatory frameworks
  ○ Enforce regulations on safety and ethics
  ○ Establish clear guidelines for advertising
  ○ Create a reporting system for adverse events
4. Addressing emerging trends
  ○ Embrace innovation responsibly
  ○ Promote evidence‐based practice
  ○ Address the impact of social media

By implementing these strategies, the field of aesthetic medicine can move toward a more regulated, standardized, and ethical landscape, benefiting both practitioners and patients.

### 5.12 Limitations of the Study

Though this study touches a very pertinent question, it carries some limitations. The sample size for one is relatively small. Questionnaires, while valuable, have limitations including the potential for biased responses, limited depth of information, and concerns about data security and privacy. Using closed‐ended questions restricts participants ability to express themselves, which may lead to misunderstanding or incomplete data.

Despite these limitations, this study does address a sensitive area, and future research may be able to build on this study to protect the ethical concerns in aesthetic medicine.

## 6 Conclusion

Aesthetic medicine and surgery have become a commodity in recent years, rather than a recognized medical specialization because of their high evolution index and high commercial incentives. From an ethical perspective, one must ask themselves if this evolution creates more problems than it solves. This study presents many reasons against this trend and demonstrates how aesthetic medicine that solely operates in accordance with market categories faces the danger of losing sight of the patients' true needs. If aesthetic medicine recognizes itself as a component of a market, it will only function as a component of the beauty business, which prioritizes profit over providing for the needs of the public. If no concrete measures are taken, aesthetic medicine is facing the threat of not being medicine anymore and becoming a beauty salon practice.

## Author Contributions

All authors contributed to data analysis, drafting or revising the article, gave final approval of the version to be published, and agree to be accountable for all aspects of the work.

## Disclosure

Two authors are consultants for Sinclair Pharma. Dr. Kamran Izhar Qureshi is a trainer for Sinclair Pharma, and Dr. Franco Vercesi is the International Key Opinion Leader for Sinclair Pharma.

## Conflicts of Interest

The authors declare no conflicts of interest.

## Supporting information


**Appendix S1.** Doctors survey results.

**Appendix S2.** General population survey results.

## Data Availability

The data that supports the findings of this study are available in the Supporting Information material of this article.
