# Supplementary material for: Are We in an Ethical Dilemma in Aesthetic Medicine?
Source: J Cosmet Dermatol. 2025 Jun 4;24(6):e70260. doi: 10.1111/jocd.70260 (PMC12136130; doi:10.1111/jocd.70260)

Appendix 1

Are we in an ethical dilemma in aesthetic medicine?
110 responses

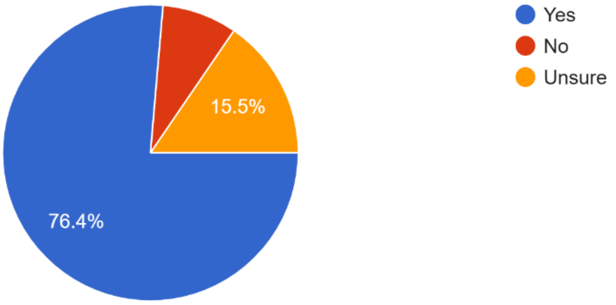

Do you think physiotherapists, pharmacists, or non-medical doctors should be allowed to perform injectables?
110 responses

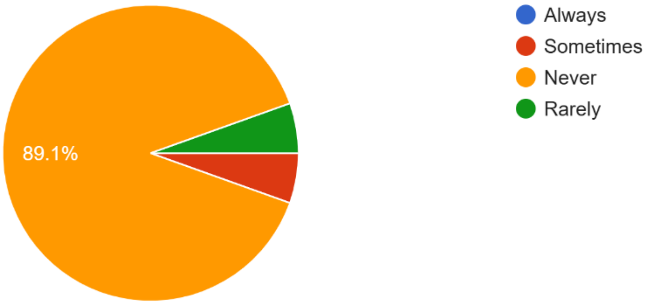

Do you think dentists should be allowed to treat skin problems and body areas other than the face?
110 responses

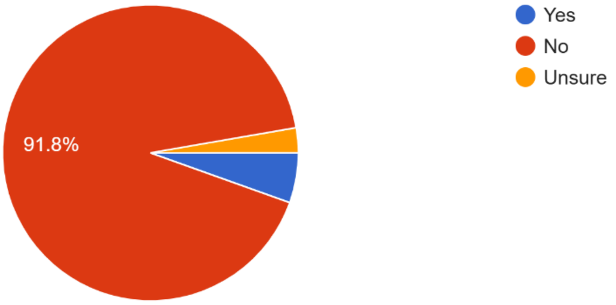

How important is it for physicians in aesthetic medicine to prioritize patient well-being over financial incentives?

110 responses

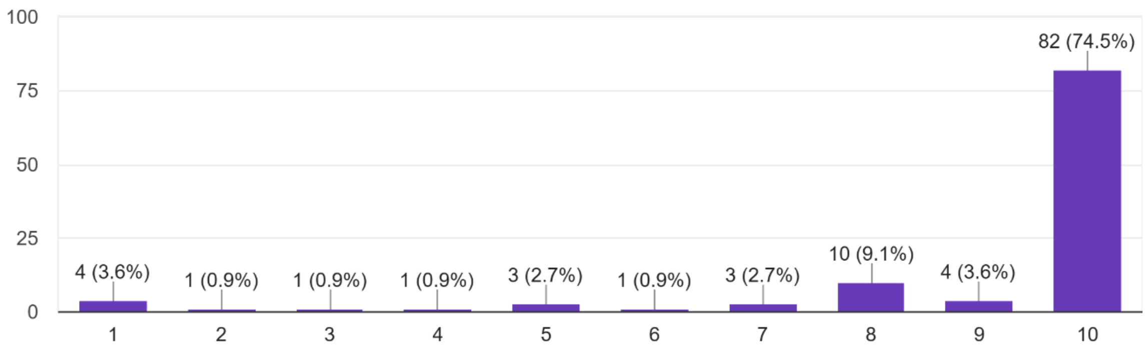

Should regulations be enforced to control how aesthetic procedures are marketed on social media?

110 responses

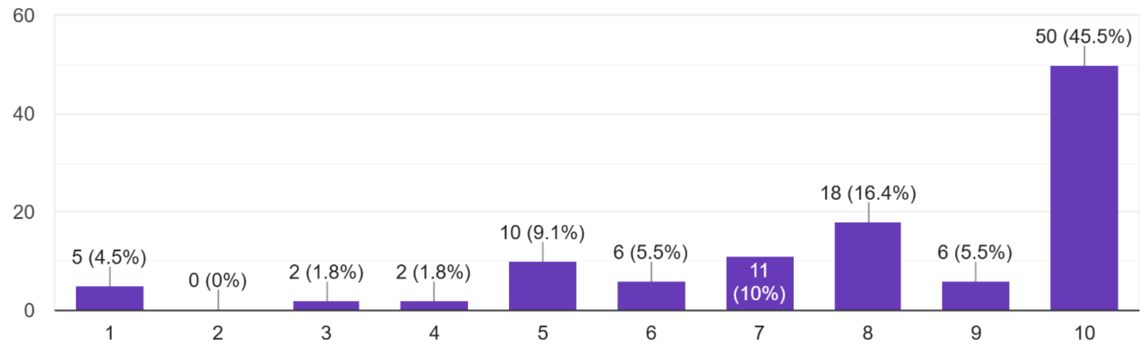

How likely are aesthetic treatments to cause long-term health risks?

110 responses

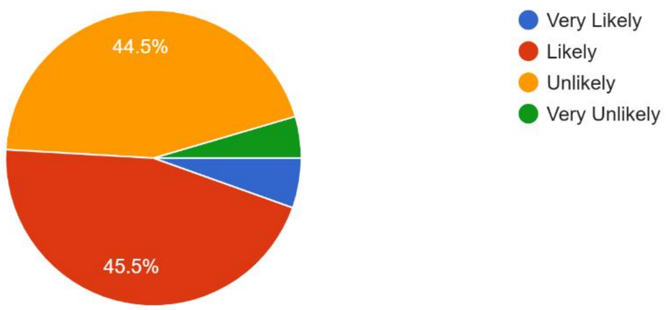

Should aesthetic procedures still follow strict medical ethics even if they do not treat an illness?

110 responses

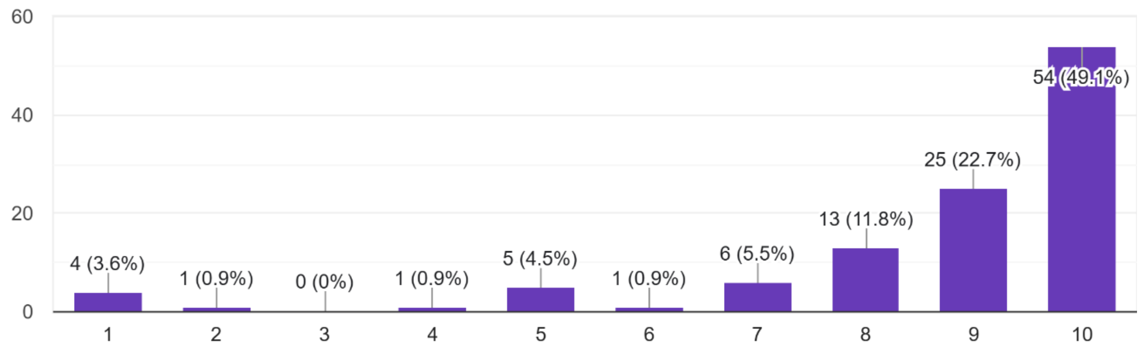

Are we following evidence-based medicine?

110 responses

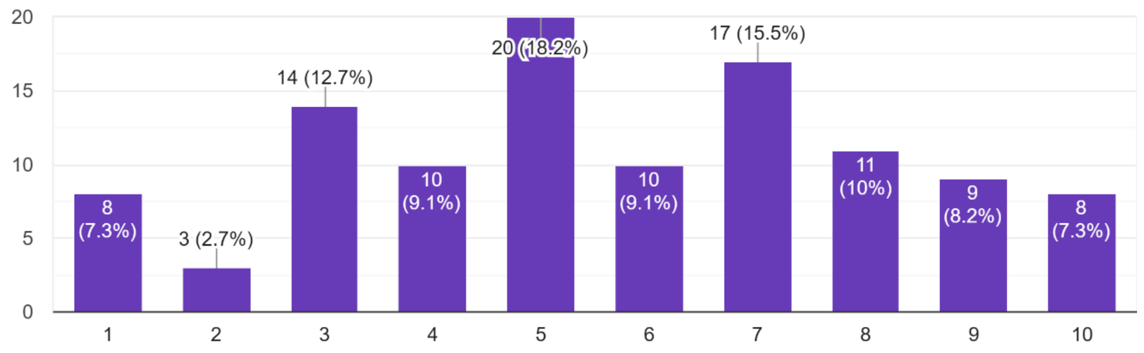

Do you believe aesthetic medicine is shifting too close to beauty salon practices?

110 responses

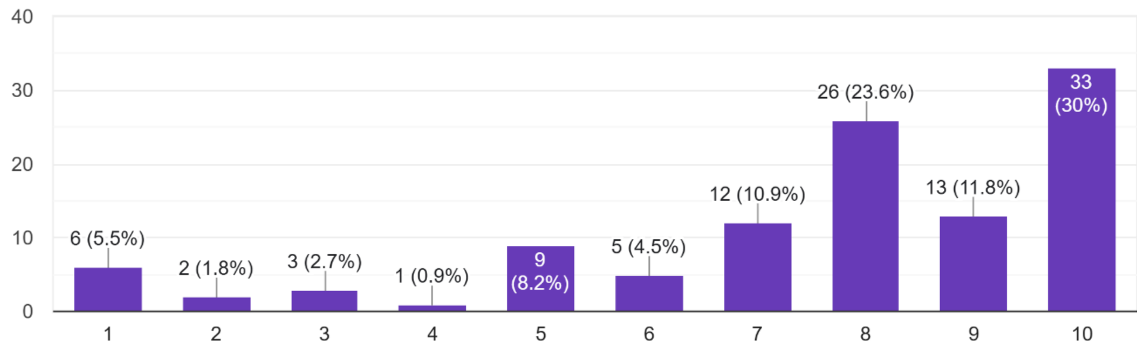

Do you feel that financial incentives in aesthetic medicine lead to overtreatment of patients?

110 responses

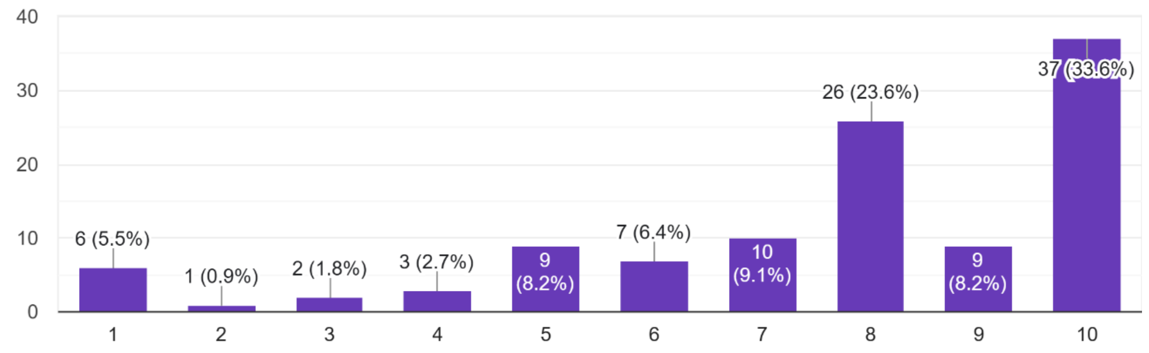

How confident are you that patients are provided with sufficient information to make informed decisions?

110 responses

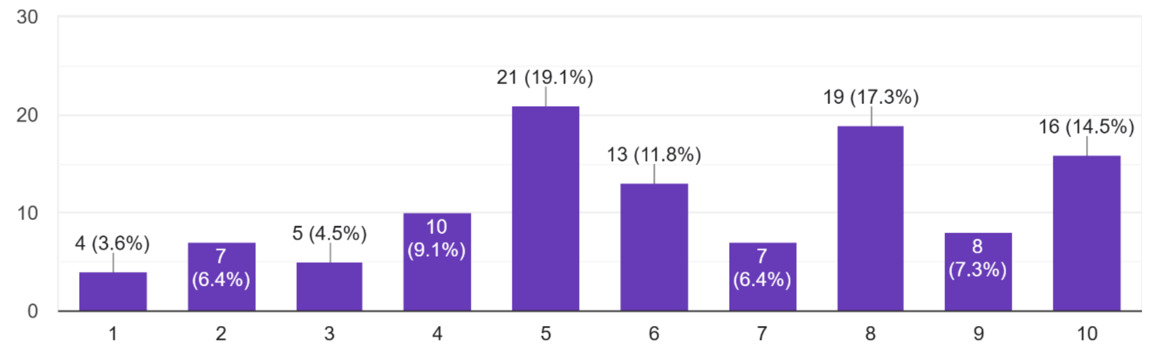

Should the responsibility lie with the doctor or patient to ensure the risks and benefits are fully understood before treatment?

110 responses

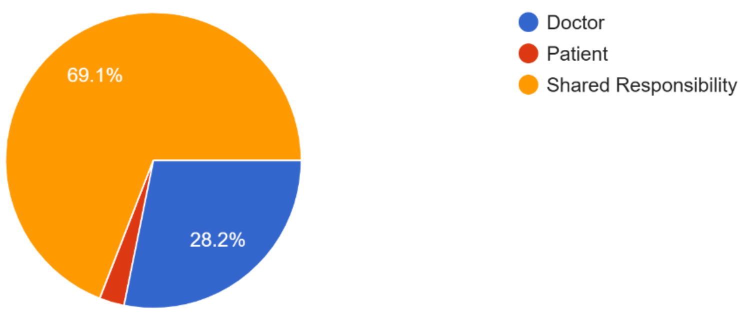

How often do you think aesthetic clinics provide accurate and unbiased information about procedures?
110 responses

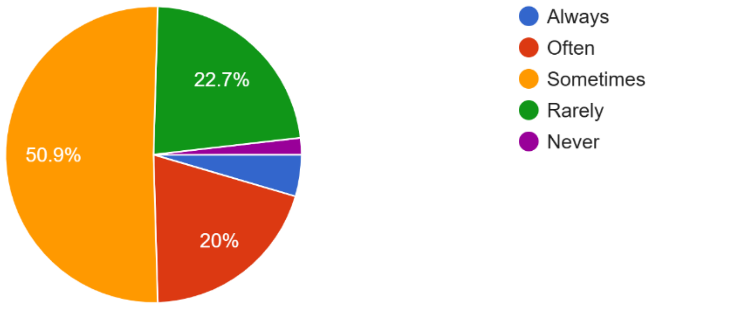

Do you think there is a race amongst clinics to be the first one to offer a procedure (Me-First Syndrome)?
110 responses

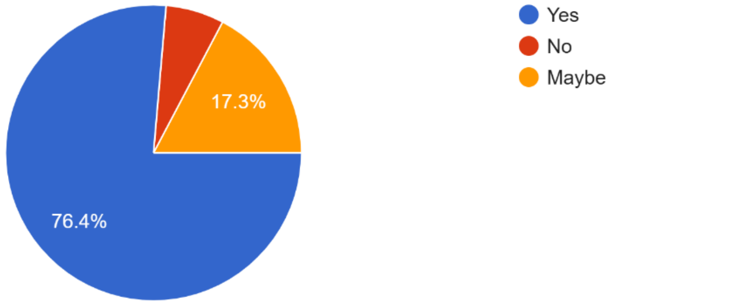

Do you believe that aesthetic practitioners are helping cure insecurities, or are they creating them?
110 responses

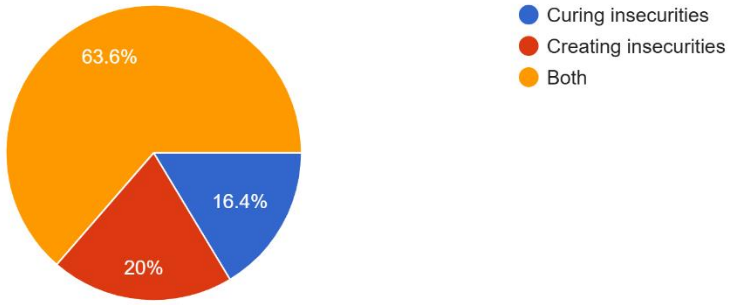

Who do you think defines modern beauty standards?

110 responses

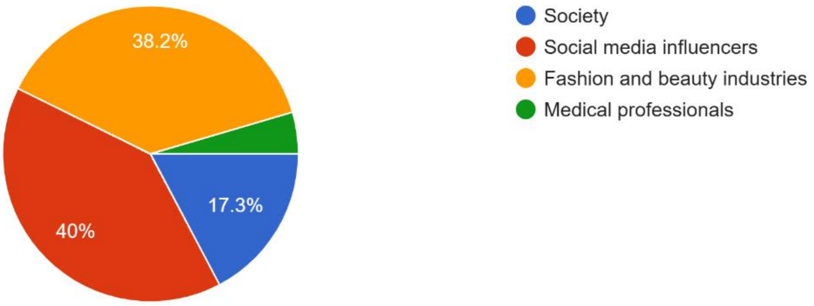

Do you think beauty trends and beauty standards are the same thing?

110 responses

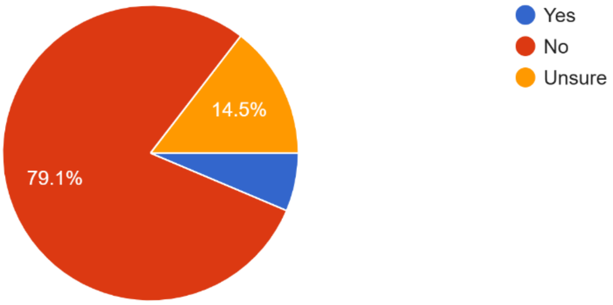

Do you think everyone can be an expert in aesthetics?

110 responses

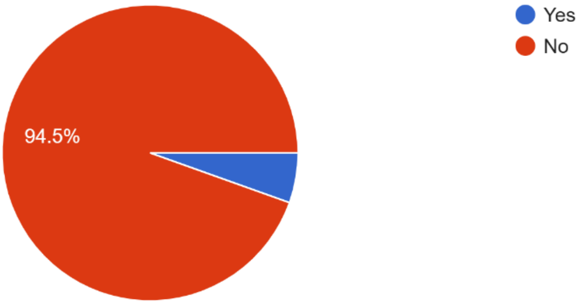

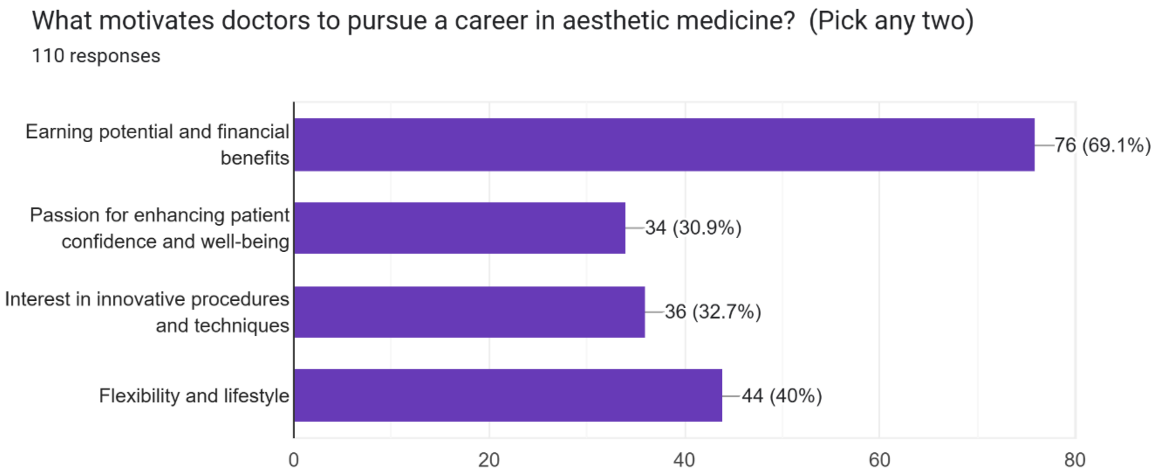
What motivates doctors to pursue a career in aesthetic medicine? (Pick any two)
110 responses
Earning potential and financial benefits
Passion for enhancing patient confidence and well-being
Interest in innovative procedures and techniques
Flexibility and lifestyle
76 (69.1%)
34 (30.9%)
36 (32.7%)
44 (40%)
0
20
40
60
80

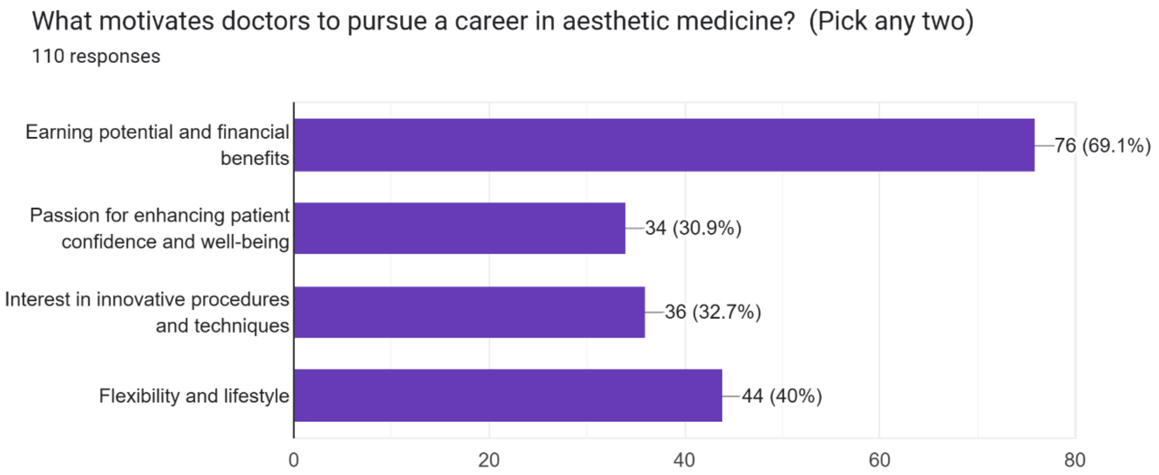
What motivates doctors to pursue a career in aesthetic medicine? (Pick any two)
110 responses
Earning potential and financial benefits
Passion for enhancing patient confidence and well-being
Interest in innovative procedures and techniques
Flexibility and lifestyle
76 (69.1%)
34 (30.9%)
36 (32.7%)
44 (40%)
0
20
40
60
80

Figure 20

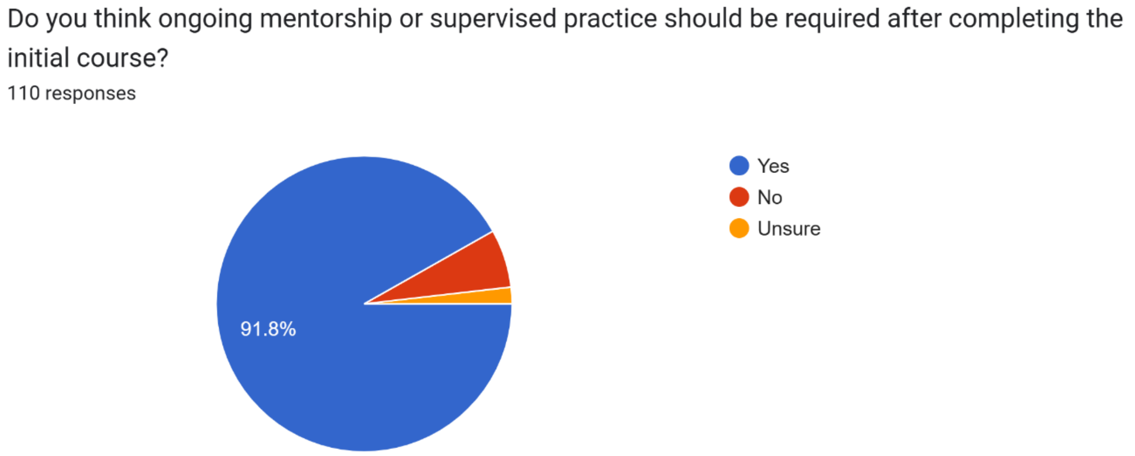
Do you think ongoing mentorship or supervised practice should be required after completing the initial course?
110 responses
91.8%
Yes
No
Unsure

How long do you believe the minimum training duration should be to safely practice aesthetic procedures?

110 responses

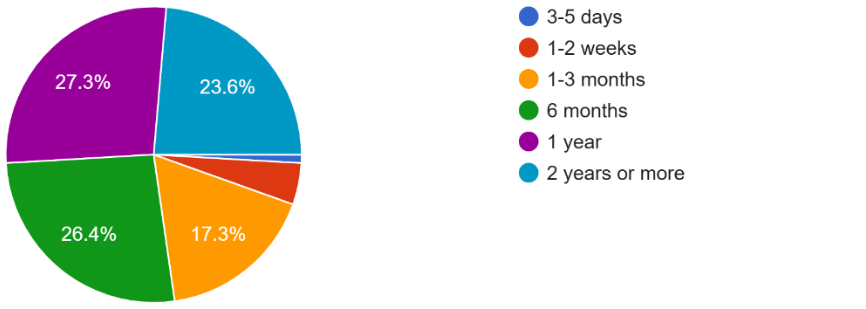

Should doctors be allowed to advertise their aesthetic services?

110 responses

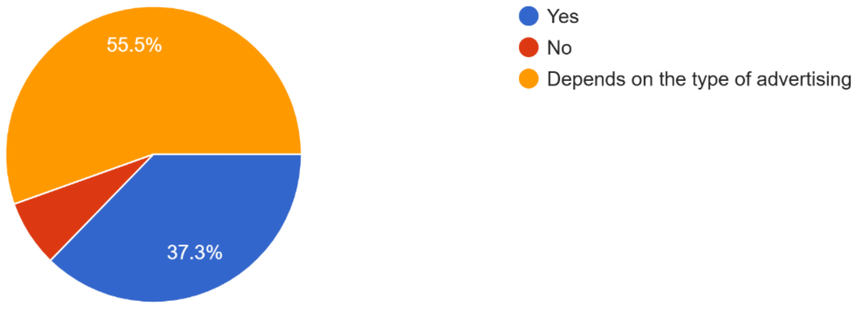

How familiar are you with Body Dysmorphic Disorder (BDD)?

110 responses

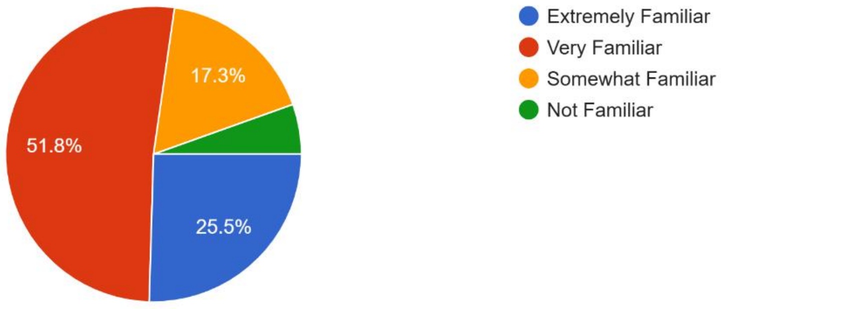

Do you think aesthetic procedures should be offered to patients diagnosed with BDD?

110 responses

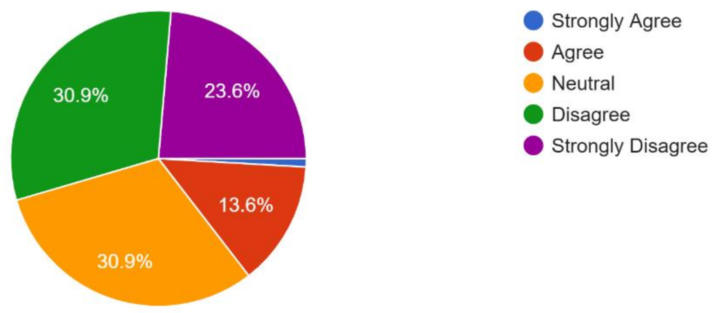

Do you think clinics should screen patients for BDD before offering aesthetic treatments?

110 responses

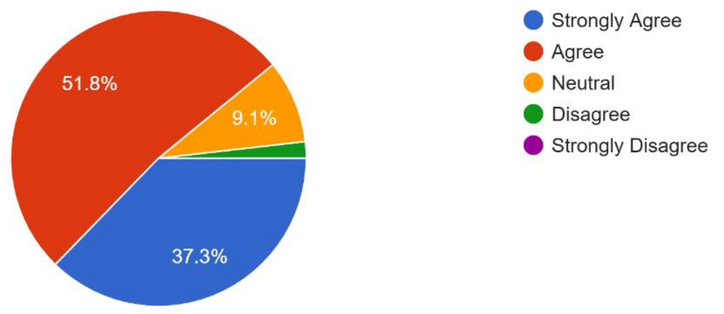

How important is specialized training in BDD for healthcare professionals offering aesthetic treatments?

110 responses

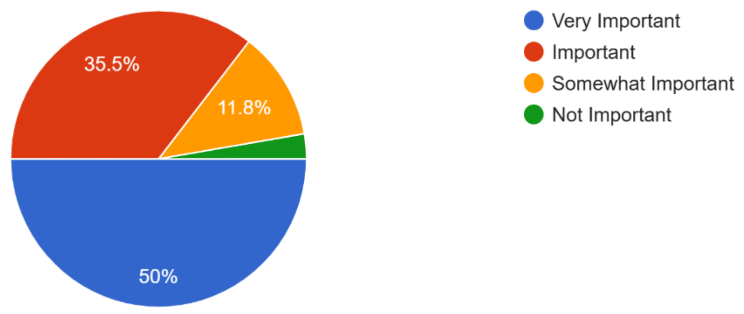

Should aesthetic medicine become a standalone medical specialty (like dermatology or plastic surgery)?

110 responses

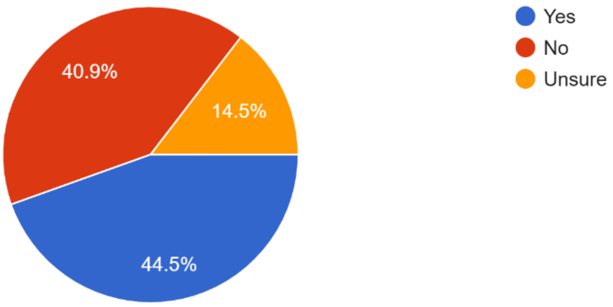

Do you feel doctors are losing their identity by giving in to social media pressures?

110 responses

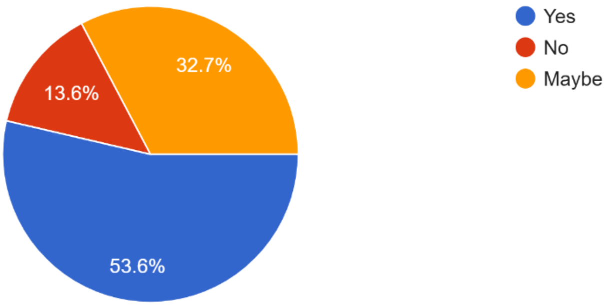

After filling this survey do you feel we are in an ethical dilemma in aesthetic medicine?

110 responses

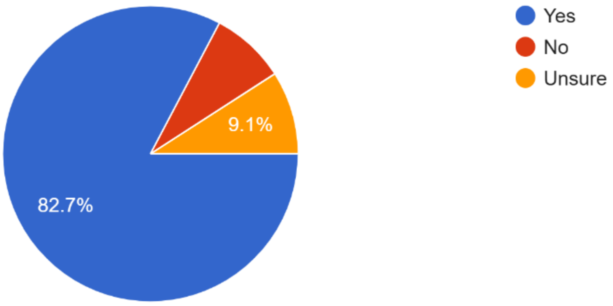

Supplement: Supplementary file 1 — Appendix S1. Doctors survey results. [file JOCD-24-e70260-s002.pdf]
