# Supplementary material for: Are We in an Ethical Dilemma in Aesthetic Medicine?
Source: J Cosmet Dermatol. 2025 Jun 4;24(6):e70260. doi: 10.1111/jocd.70260 (PMC12136130; doi:10.1111/jocd.70260)

Appendix 2: Questionnaire Results – General Population

Ethnicity

114 responses

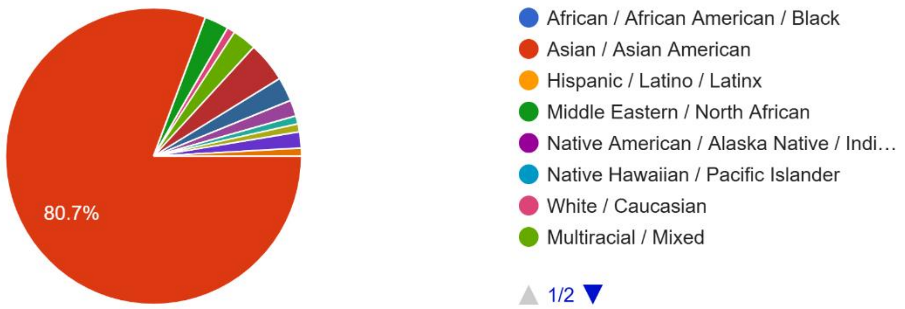

Age

114 responses

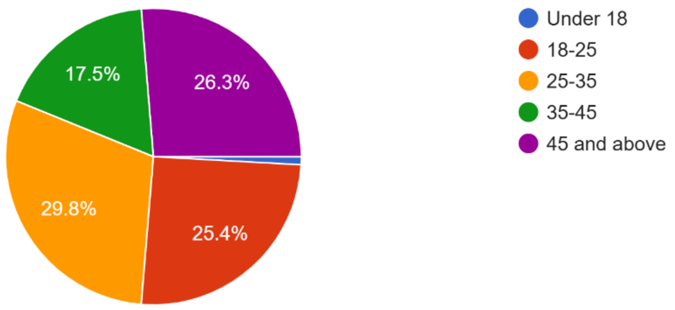

Are we in an ethical dilemma in aesthetic medicine?

114 responses

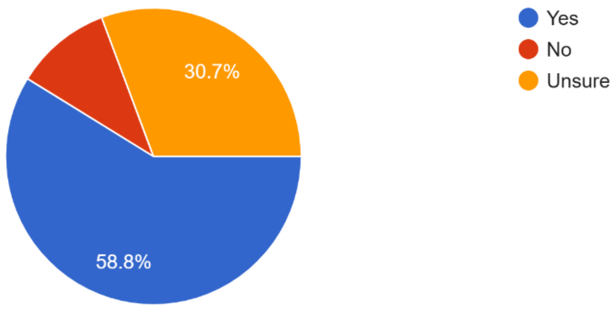

Do you think physiotherapists, pharmacists, dentists or non-medical doctors should be allowed to perform injectables?

114 responses

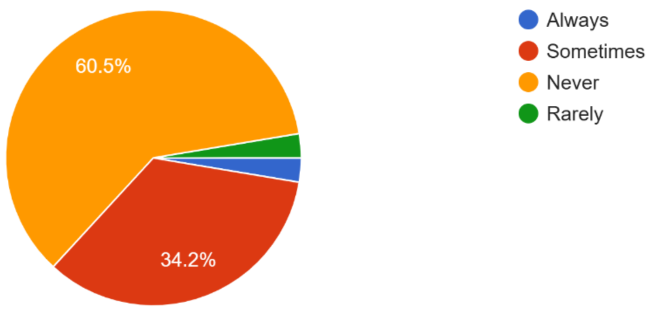

Do you think dentists should be allowed to treat skin problems and body areas other than the face?

114 responses

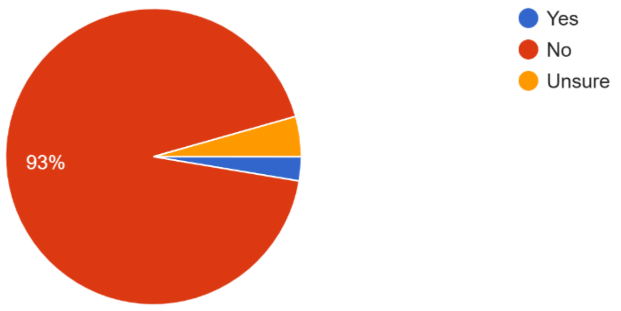

Do you think there is a race amongst clinics to be the first one to offer a procedure (Me-First Syndrome)?

113 responses

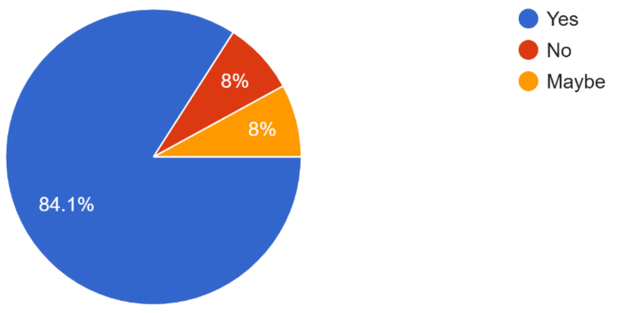

How important is it for physicians in aesthetic medicine to prioritize patient well-being over financial incentives?

114 responses

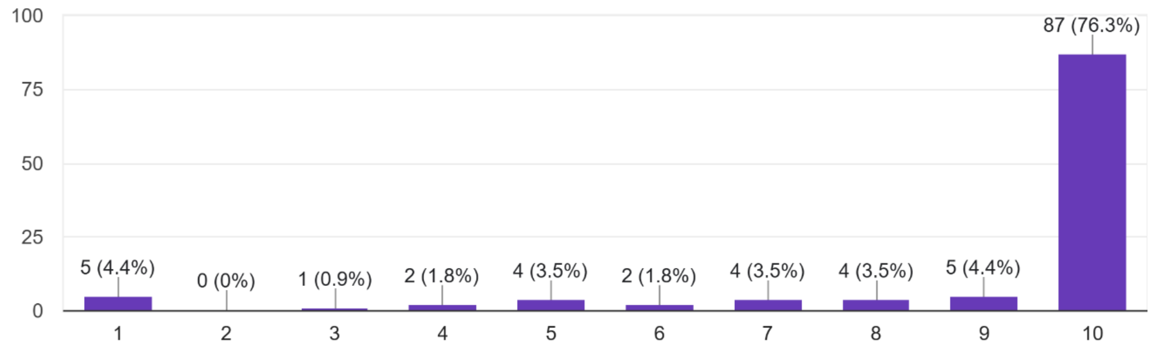

Do you believe "before-and-after" images used in marketing can create unrealistic expectations?

114 responses

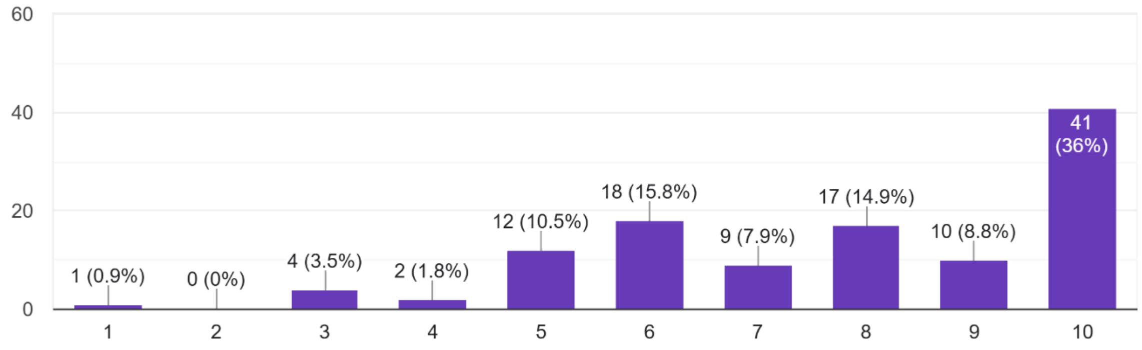

Should regulations be enforced to control how aesthetic procedures are marketed on social media?

114 responses

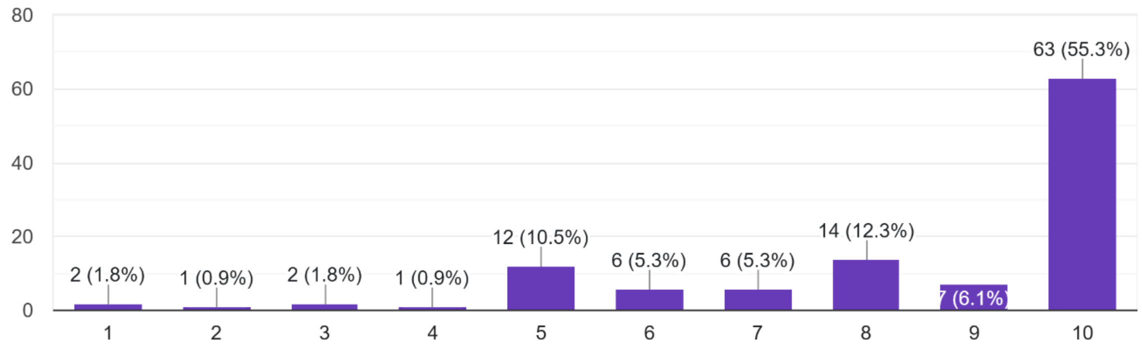

How likely are aesthetic treatments to cause long-term health risks?

114 responses

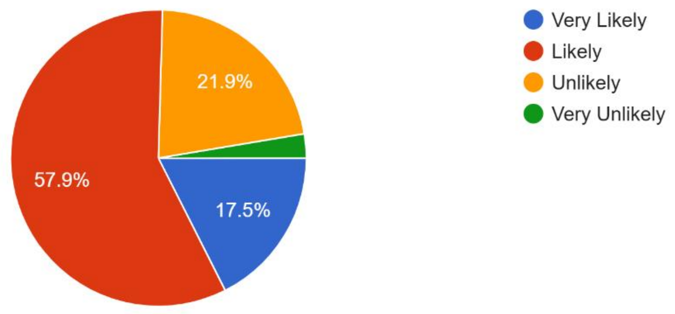

Should aesthetic procedures still follow strict medical ethics even if they do not treat an illness?

114 responses

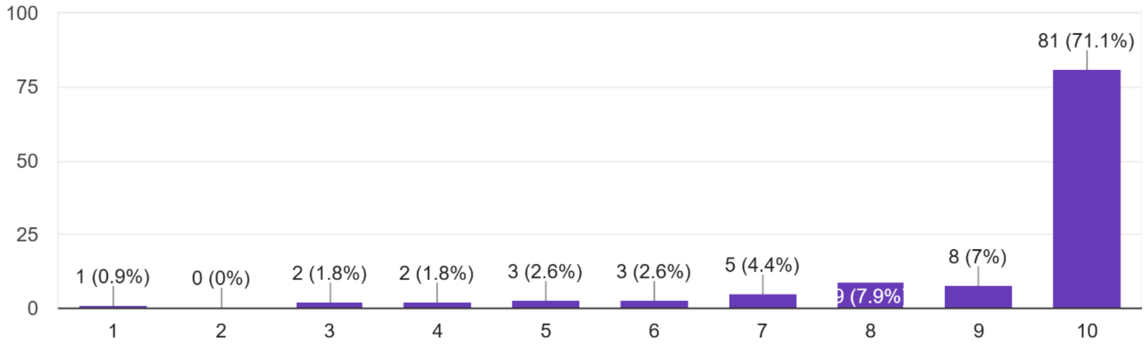

Are doctors following evidence-based medicine?

114 responses

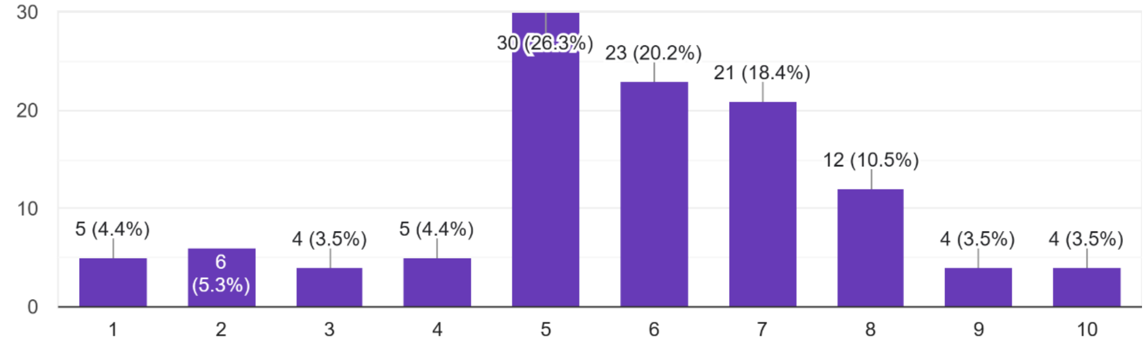

Do you believe aesthetic medicine is shifting too close to beauty salon practices?
114 responses

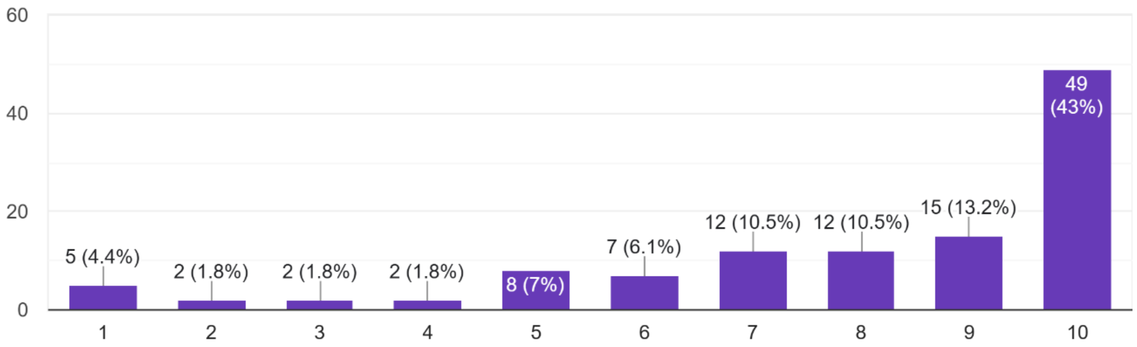

Do you feel that financial incentives in aesthetic medicine lead to overtreatment of patients?
114 responses

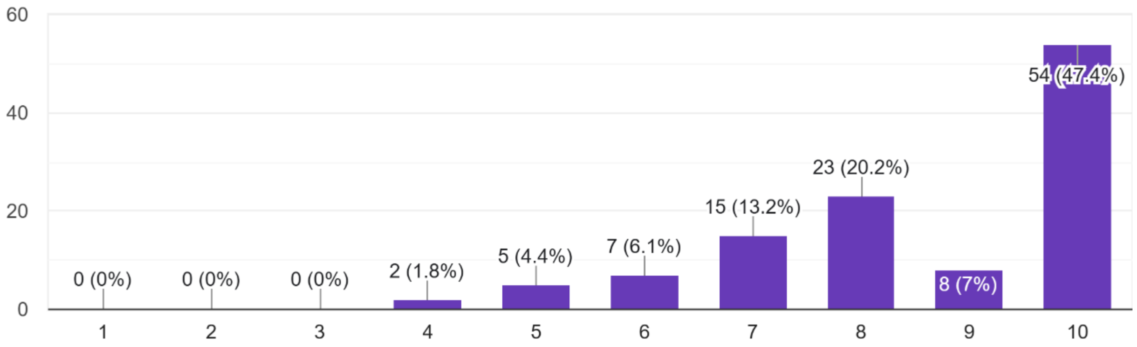

How likely are you to trust aesthetic clinics that heavily market their services on social media?
114 responses

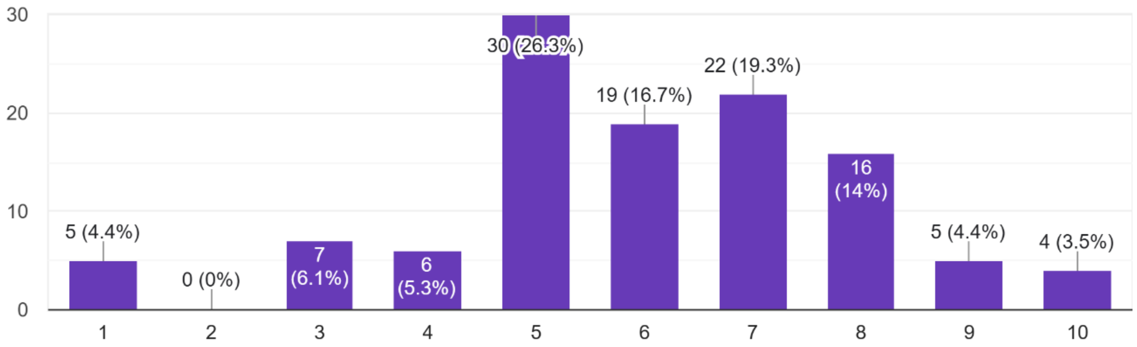

How confident are you that patients are provided with sufficient information to make informed decisions?

114 responses

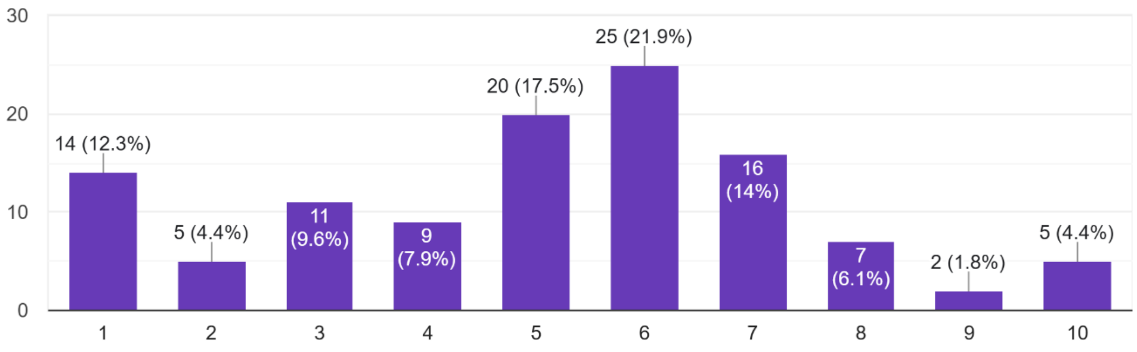

Should the responsibility lie with the doctor or patient to ensure the risks and benefits are fully understood before treatment?

114 responses

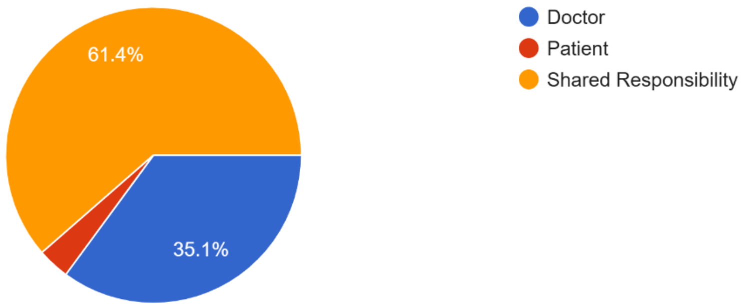

How often do you think aesthetic clinics provide accurate and unbiased information about procedures?

114 responses

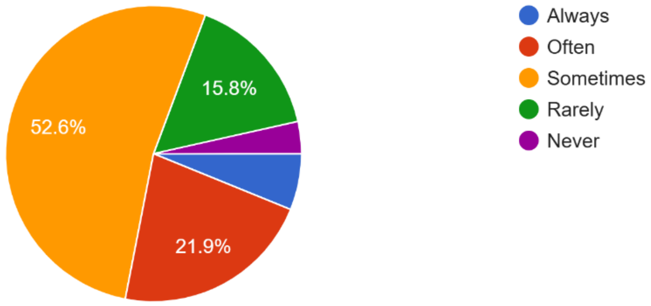

What do you look at before selecting a clinic?

114 responses

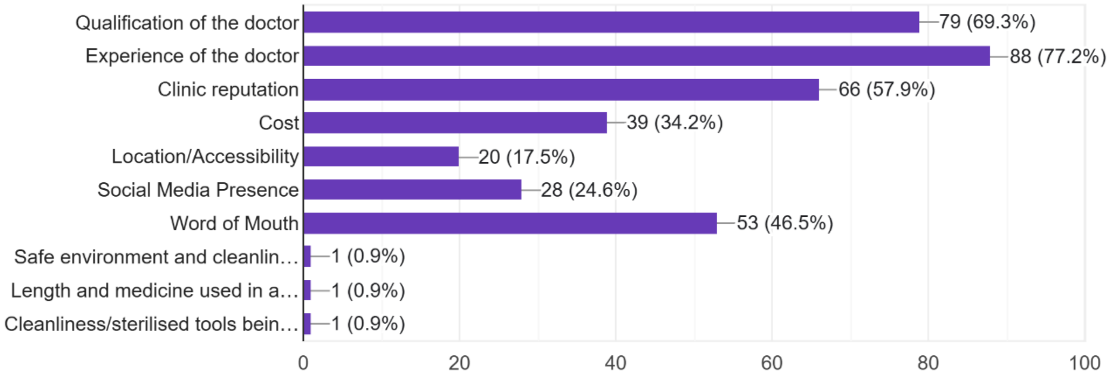

Do you consider the doctor's experience important in your selection?

114 responses

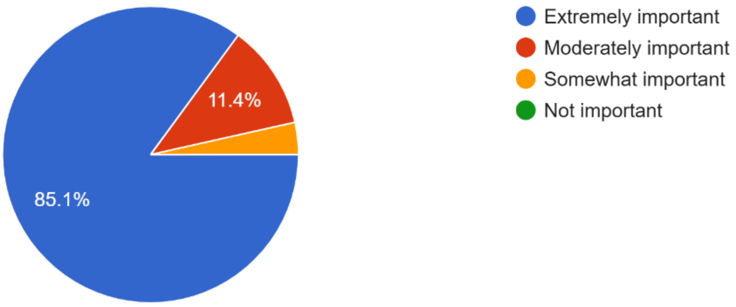

Do you look at the qualifications of the doctor before selecting a clinic?

114 responses

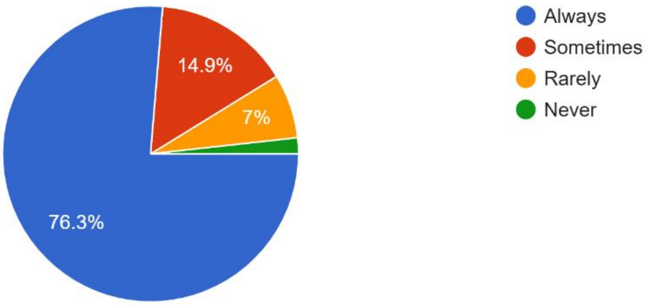

Are you aware that not all practitioners in aesthetic medicine are medical doctors?
114 responses

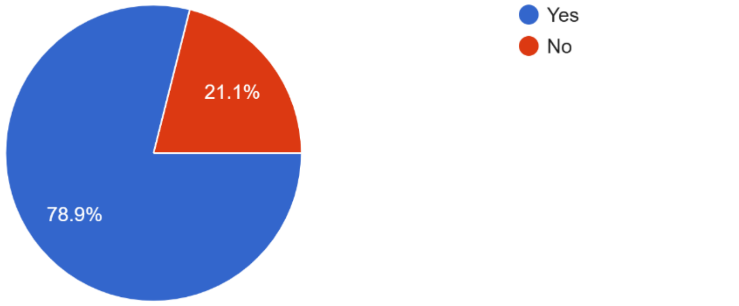

How important is it to you that your practitioner is a qualified medical doctor?
114 responses

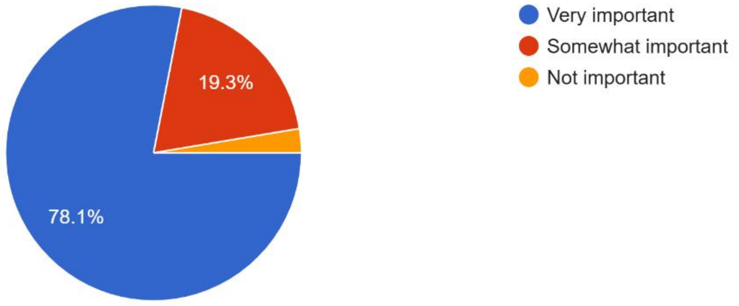

Is cost the main factor in choosing an aesthetic clinic?
114 responses

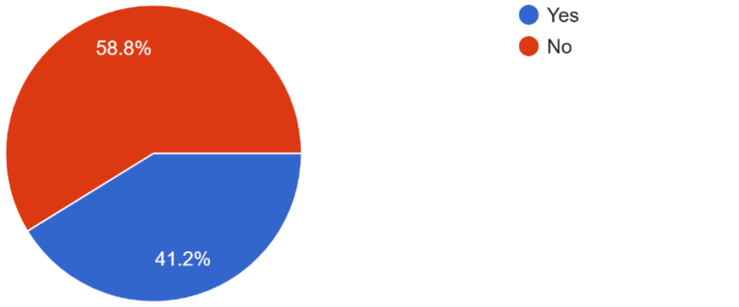

Do you ask the practitioner about the procedure and products being used?

114 responses

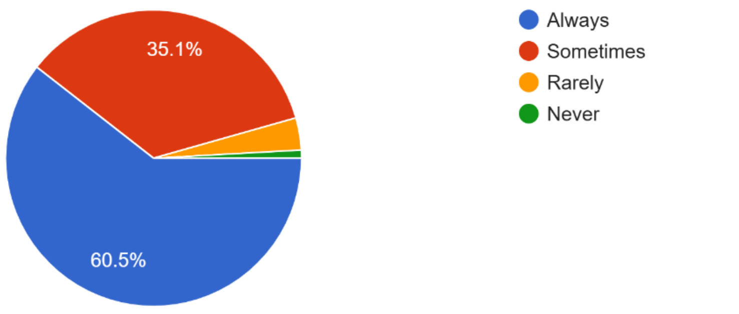

Do you believe that aesthetic practitioners are helping cure insecurities, or are they creating them?

114 responses

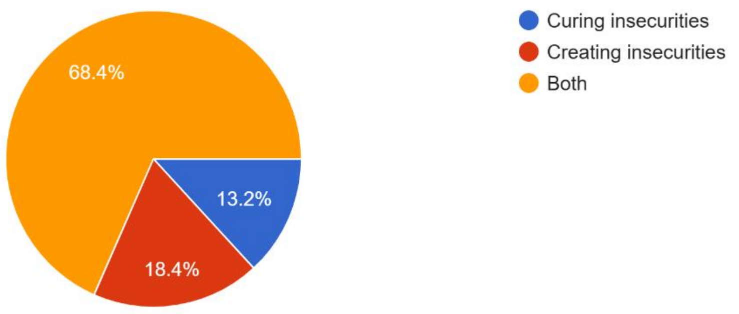

On a scale of 1 to 10, how much do you believe what you see on social media?

114 responses

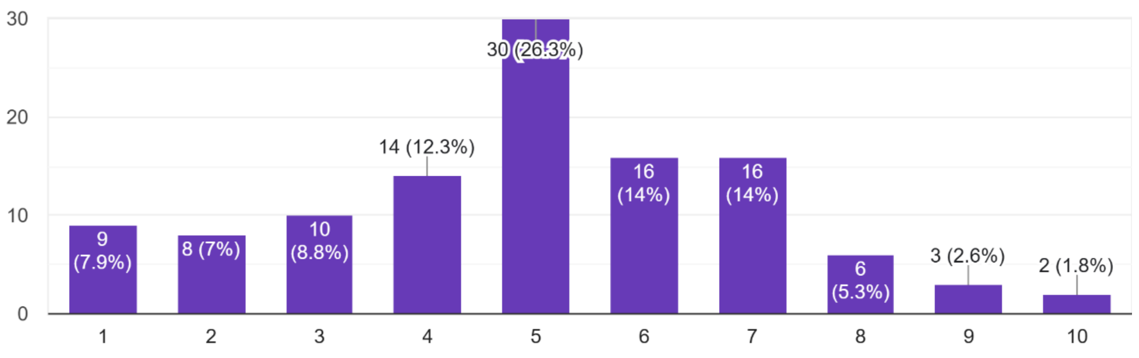

Do you believe that results seen on someone else can be reproduced on you?

114 responses

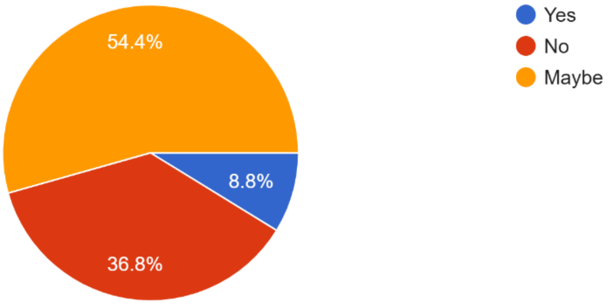

Who do you think defines modern beauty standards?

114 responses

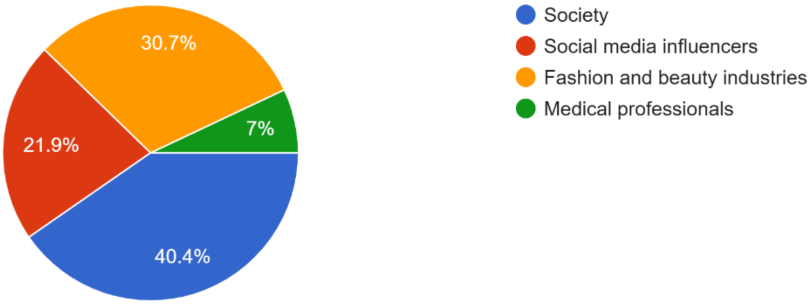

Do you think beauty trends and beauty standards are the same thing?

114 responses

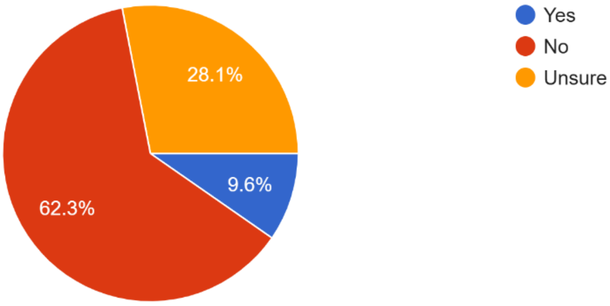

Do you think everyone can be an expert in aesthetics?

114 responses

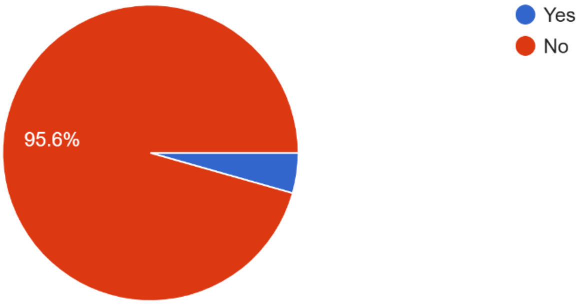

How much do you trust your aesthetic practitioner?

114 responses

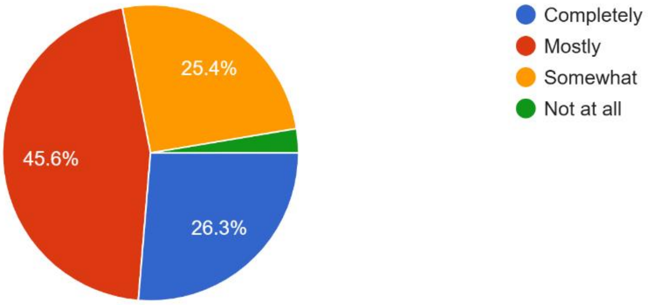

Should doctors be allowed to advertise their aesthetic services?

114 responses

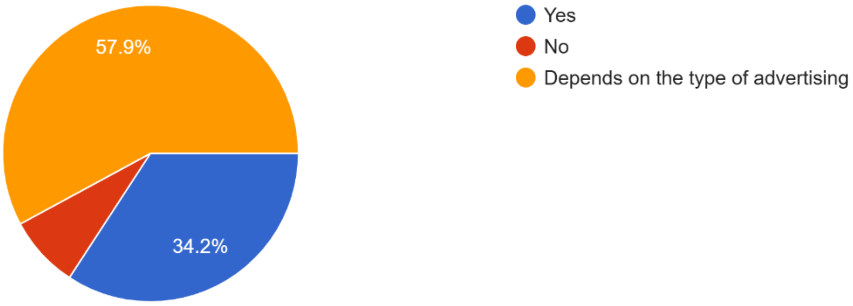

How familiar are you with Body Dysmorphic Disorder (BDD)?
114 responses

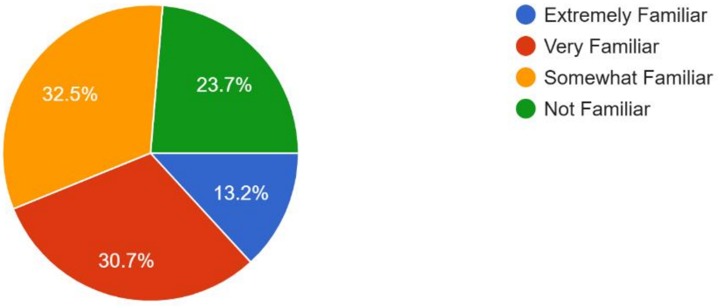

Do you think aesthetic procedures should be offered to patients diagnosed with BDD?
114 responses

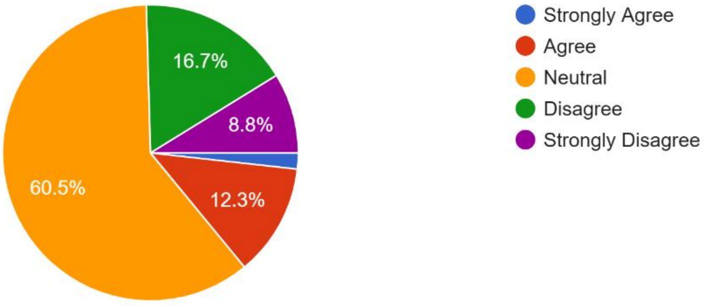

Do you think clinics should screen patients for BDD before offering aesthetic treatments?
114 responses

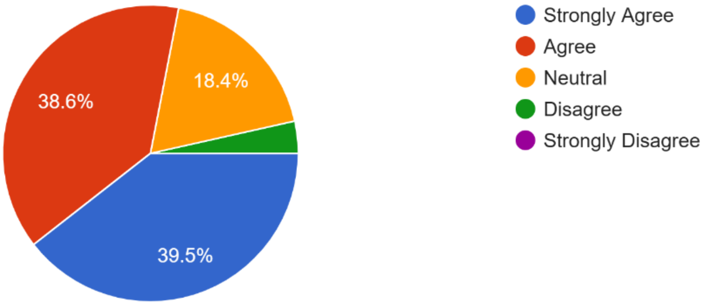

How important is specialized training in BDD for healthcare professionals offering aesthetic treatments?

114 responses

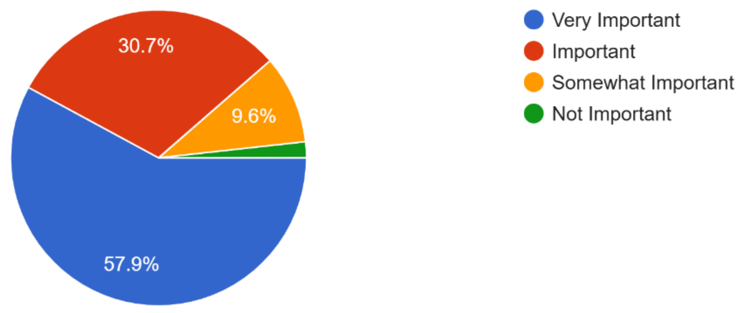

Do you edit or apply filters to your photos before posting on social media?

114 responses

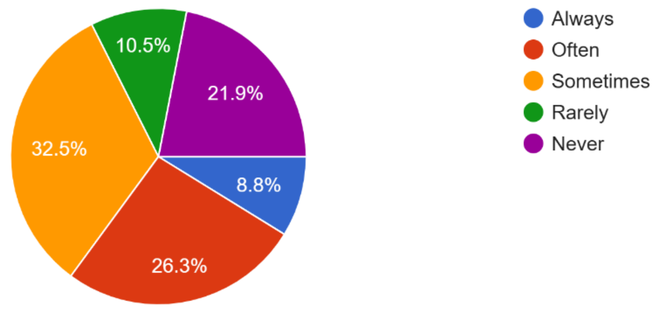

Do you feel social media pressures you to alter your appearance or use filters?

114 responses

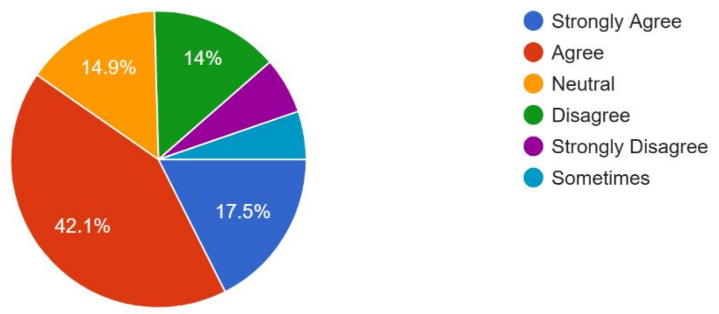

How likely are you to try a new aesthetic service offered by a clinic?

114 responses

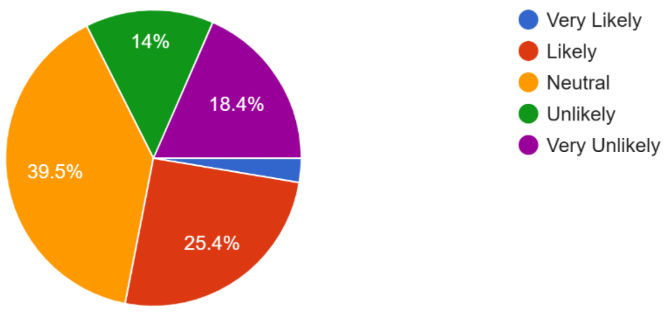

What factors influence your interest in a new offering from an aesthetic clinic? (Select all that apply)

114 responses

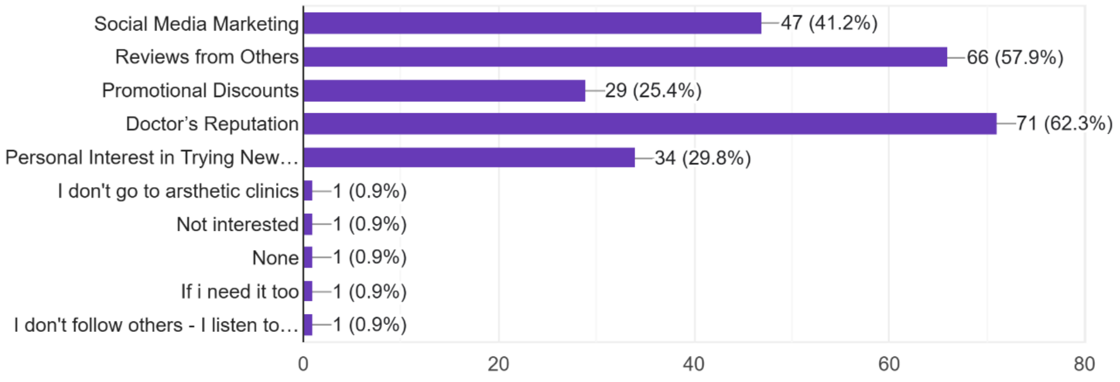

What attracts you the most when you see the content on the social media of an Aesthetic Clinic?

114 responses

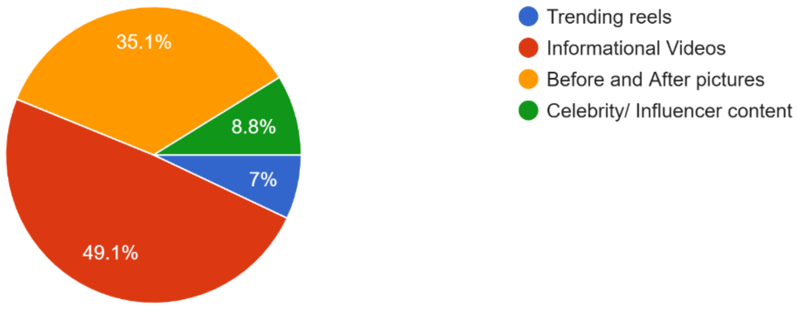

After filling this survey do you feel we are in an ethical dilemma in aesthetic medicine?

114 responses

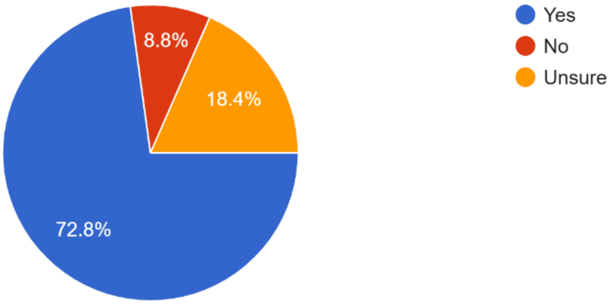

Do you feel doctors are losing their identity by giving in to social media pressures?

114 responses

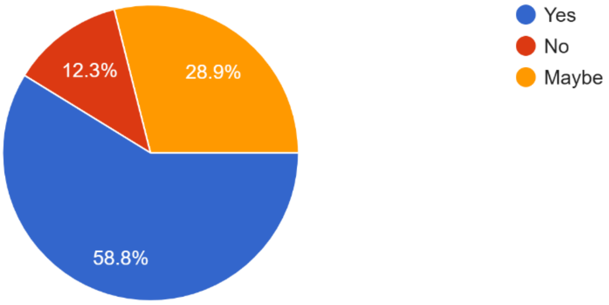

Supplement: Supplementary file 2 — Appendix S2. General population survey results. [file JOCD-24-e70260-s001.pdf]
